# Participation and Contribution in Crowdsourced Surveys

**DOI:** 10.1371/journal.pone.0120521

**Published:** 2015-04-02

**Authors:** Robert Swain, Alex Berger, Josh Bongard, Paul Hines

**Affiliations:** 1 Computer Science Department (graduated), University of Vermont, Burlington, United States of America; 2 School of Business Administration, University of Vermont, Burlington, United States of America; 3 Computer Science Department, University of Vermont, Burlington, United States of America; 4 School of Engineering, University of Vermont, Burlington, United States of America; 5 Complex Systems Center, University of Vermont, Burlington, United States of America; Örebro University, SWEDEN

## Abstract

This paper identifies trends within and relationships between the amount of participation and the quality of contributions in three crowdsourced surveys. Participants were asked to perform a collective problem solving task that lacked any explicit incentive: they were instructed not only to respond to survey questions but also to pose new questions that they thought might-if responded to by others-predict an outcome variable of interest to them. While the three surveys had very different outcome variables, target audiences, methods of advertisement, and lengths of deployment, we found very similar patterns of collective behavior. In particular, we found that: the rate at which participants submitted new survey questions followed a heavy-tailed distribution; the distribution in the types of questions posed was similar; and many users posed non-obvious yet predictive questions. By analyzing responses to questions that contained a built-in range of valid response we found that less than 0.2% of responses lay outside of those ranges, indicating that most participants tend to respond honestly to surveys of this form, even without explicit incentives for honesty. While we did not find a significant relationship between the quantity of participation and the quality of contribution for both response submissions and question submissions, we did find several other more nuanced participant behavior patterns, which did correlate with contribution in one of the three surveys. We conclude that there exists an optimal time for users to pose questions early on in their participation, but only after they have submitted a few responses to other questions. This suggests that future crowdsourced surveys may attract more predictive questions by prompting users to pose new questions at specific times during their participation and limiting question submission at non-optimal times.

## Introduction

Crowdsourcing [1] holds that a large group of non-experts can each contribute a small amount of effort to solve a problem that would otherwise require a large amount of effort from a smaller, expert group to complete. Popular examples of crowdsourcing include the online encyclopedia Wikipedia [2], the news aggregation site Reddit.com [3], and Amazon’s Mechanical Turk [4]. Crowdsourcing has been employed to help solve a wide variety of tasks. Amazon’s Mechanical Turk has been used to crowdsource data annotations [5], behavioral research [6], assessment of visualization design [7], human language technologies [8], and transcribing audio [9]. Other stand-alone websites have been used to crowdsource everything from mapping the aftermath of the 2010 earthquake in Haiti [10] to predicting protein structures [11]. In each of these examples, some crowdsourcing participants participate more than others, while some participants produce better quality contributions. Several studies [12–14] have shown that participation per user follows a power law relationship, but the quality of that participation has not been fully explored. In this paper, we use the results of three crowdsourcing studies to examine the relationship between participation rates and quality of contribution.

Due to the fact that work is distributed among a large group of participants [15], and coupled with the fact that many crowdsourcing efforts are voluntary and do not compensate participants explicitly [16], participation tends to be sporadic and non-uniformly distributed among participants. As seen in numerous crowdsourcing studies, participation tends to follow a power law distribution in which a small number of users participate a great deal, and the vast majority of users participate very little [17]. In fact, this trend has been modeled explicitly in some of the largest examples of crowdsourcing. Wilkinson [12] showed that user participation in four popular crowdsourcing websites (Wikipedia, Digg, Bugzilla, Essembly) all follow a power law distribution with the power law exponents found to be “strongly related to the system’s barrier to contribution” (p. 3). Wu *et al.*[13] observe that user contribution on the sites Youtube and Digg also follow a power law. They find that the attention and popularity of users’ contributions heavily influence a user’s probability of continuing to contribute. This results in a “rich get richer” feedback loop of attention focused on a core group of power users. In a similar study involving the popular South Korean question/response site “Naver Knowledge-iN”, Nam *et al.*[14] also found that user participation resulted in a power law distribution.

Looking beyond raw participation, a few studies have examined the quality of contributions in crowdsourcing applications. Using Amazon’s Mechanical Turk platform, several studies have found that monetary incentives do not significantly impact the quality of contributions [18, 19, 20, 21, 22]. Downs *et al.*[23] found that some users contribute a large quantity of low quality content in order to earn more financial reward. Chandler and Kapelner [24] found that by framing a crowdsourcing task to make it seem more meaningful to the participant resulted in increased quality of contributions.

In this paper, we study the relationship between participation and contribution using the results from three experiments involving the crowdsourcing of surveys. While these three experiments focused on very different subject domains (health, electricity usage, and financial behavior), each used the same essential crowdsourcing algorithm, which is described under Methods.

## Methods

The crowdsourcing method employed in this paper was motivated by the hypothesis that there are many questions of scientific and societal interest, for which the general (non-expert) public has substantial insight. For example, patients who suffer from a disease, such as diabetes, are likely to have substantial insight into their condition that is complementary to the knowledge of scientifically-trained experts. The question is, how does one collect and combine this non-expert knowledge to provide scientifically-valid insight into the outcome of interest.

To address this question, we developed and tested a crowdsourced survey method (reported on in prior work [25, 26]), which proceeds approximately as follows. After being invited to contribute, participants begin by answering online survey questions in the traditional manner. In addition, they are invited to inject questions of their own into the survey that they believe may be predictive of an outcome variable of interest. This results in the number of questions in the survey growing over time at a non-uniform rate. As new questions are added to the survey, subsequent participants have the opportunity to answer them. Finally, a real-time machine learning module continuously builds models from this growing store of data to predict the outcome variable, and the resulting predictions are shown to the users.

For this paper we study data from three separate instantiations of this concept. In the first study, participants were asked to pose and answer questions ab out their childhood that could predict their current, adult Body Mass Index (Childhood BMI [26]). In the second study, participants were asked to pose and answer questions that could predict their monthly home electricity usage (EnergyMinder). In the third study, participants were asked to pose and answer questions that might predict their primary bank account balance (Personal Savings).

In the remainder of this section we describe the crowdsourcing algorithm and implementations in detail, and provide details on the methods used in our data analysis.

### Crowdsourcing Algorithm

**Fig 1:**
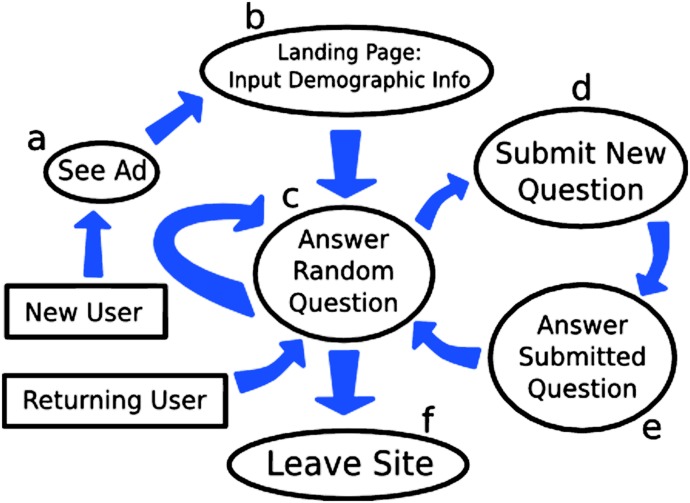
The Crowdsourcing Algorithm. Illustration of the crowdsourced survey algorithm used in all three of the studies in this paper. The figure shows an average use case for a typical participant.

The crowdsourcing algorithm used in the three studies examined in this paper is illustrated in Fig. 1. This process typically begins with a new user encountering an advertisement that was placed on social media by the research team (Fig. 1a). Once the new user has seen and clicked on the advertisement, they are brought to the landing page of the crowdsourcing website (Fig. 1b). The landing page displays information about the project and what participation entails, as well as an option to create login information.

If the user decides to proceed past the landing page, they enter into the survey itself (Fig. 1c). On this page the user is presented with a question from the survey. For each of the three experiments analyzed in this paper, the survey questions were presented in a uniformly random manner. In addition to being presented with a question, this page also includes a prompt for the user to add a question of their own to the survey. If the user has an idea for a new survey question, they can click the prompt and are taken to the question submittal page (Fig. 1d).

If the user decides to submit their own question, they are then required to respond to it (Fig. 1e). We have found that being forced to immediately respond to the question helps users to recognize when their questions are unclear or confusing. After the user has submitted a question, the question then enters an approval queue to wait for a survey administrator to approve the question for addition to the survey. In this way, users may pose and respond to questions in any order. In the implementations described here users were allowed to pose as many questions as they wish.

### Implementation of the Three Crowdsourced Surveys

The first deployment that we examine in this paper was a survey designed to crowdsource childhood predictors of adult BMI (Childhood BMI, [26]). The project ran from 11/8/2012 to 11/19/2012, and was advertised on three Reddit.com subreddits (r/loseit, r/keto, r/parenting), and on the Facebook profiles of the researchers. Participation was entirely voluntary with no compensation offered. Three questions seeded the study: “When you were a child, did you own a bike?”, “When you were a child, how many times did you eat at a fast food restaurant?”, and “When I was a child, I was bullied.”

Once participants clicked on the advertisement, they arrived at the landing page (S1 File), which asked users to input height, weight, age, gender, and country of origin. Inputs could be entered in US units or metric units. These fields were mandatory and participants were unable to proceed further into the site without these completed. Included on this landing page was a video describing the purpose and function of the site, a written statement detailing the purpose of the study, a statement outlining the risks of participation, and contact information.

Participants who passed the landing page came to the primary survey page (S1 File). On this page, users were shown a randomly selected question that they had not yet answered. In addition to being shown a question, users could also see their weight and BMI, the weight and BMI that the website predicted for them, and a histogram of all users’ BMIs. Just below the randomly selected question, users were prompted to add their own questions to the survey. If a user clicked on the question submittal prompt, they were taken to the question submittal page (S1 File). On this page, users were shown information about what types of questions were not allowed (self-identifying information and offensive questions), as well as examples of clear, specific questions. In order to automate the processing of the survey, we asked participants to choose from among three types of question (yes/no, numeric entry, and Likert scale).

The second deployment of the crowdsourcing algorithm was a project designed to examine the relationship between lifestyle choices and monthly home electricity use (EnergyMinder). The site is still live as of the writing of this paper (http://energyminder.net/), however for the purposes of this paper, we only consider data accumulated from the start of the project in July 2013 through March 2014. This project was undertaken in partnership with Burlington Electric Department (BED) and was available only to BED customers with smart meters installed in their homes. The number of eligible customers was approximately 24,500 (accuracy is limited due to customers moving in and out of BED’s range of supply). Advertisement was achieved via two direct mailings sent out by BED. Six questions seeded the study: “I generally use air conditioners on hot summer days”, “Do you have a hot tub?”, “How many teenagers are in your home?”, “How many loads of laundry do you do per week?”, “Do you have an electric hot water tank?”, and “Most of my appliances (laundry machines, refrigerator, etc.) are high efficiency.”

The advertisements sent out to BED customers included a brief description of the project, the project URL, and personalized login information. BED customers that chose to go to the URL came to the landing page (S1 File). This page contains fields for registering for the site, a video explaining the purpose and function of the site, as well as links to general information and frequently asked questions. Once the user registers for the site, they are taken to the primary page of the site which displays a random question to be answered (S1 File). In addition to displaying a question, this page displays a histogram of all users’ electricity usage and prompts users to submit their own question. If users decide to submit a question of their own, they can click on a link that takes them to the question submittal page (S1 File). Similar to the Childhood BMI question submittal page, this page displays guidelines for good questions, examples of poor questions, and allows for the submission of yes/no, numeric entry, and Likert scale questions.

This site was the most professional looking of the three that we examine in this paper. In contrast to the two other websites, professional website developers were hired to construct this site. This resulted in a cleaner, more modern feeling to the website. Many elements of the site were more responsive and dynamic than the other two survey websites. Whether this affected the amount and quality of participation is currently unknown.

The third application of the crowdsourcing algorithm examined here is a project designed to crowdsource hypotheses surrounding the issue of financial behavior (Personal Savings). Specifically, this project asked participants to generate questions predictive of how much money they currently have in their primary bank account. This project ran from January 2014 to February 2014. Advertising for the project was done through several Reddit.com subreddits and participation was open to anyone. Unique to this project was that a popular blogger found the site on Reddit and advertised it on their blog, which resulted in a dramatic increase in participation. The look of this site was simple and slightly less professional than the other two sites included in this paper. One question seeded the study: “What is your age?”

The landing page for this personal savings site (S1 File) consisted of a video explaining the purpose and function of the site, and a link to proceed. Upon clicking the link, participants were taken to a second landing page (S1 File) that asked users “How much money do you have in your bank account?”. Once a participant entered an amount and clicked the link to proceed, they then moved on to the primary survey page (S1 File). This page displayed a random question to be answered, the website’s current prediction of the amount of money in their bank account, as well as a prompt to submit a question of their own. For this study, users could choose to submit yes/no, numeric entry, or custom scale questions.

### Data Modeling

Data modeling methods were used in three different contexts for this paper. Within each site we used fairly simple statistical algorithms to identify relationships between the outcome of interest (BMI, energy, finance), and answers provided to user-generated questions. For the Childhood BMI and EnergyMinder sites we used a simple stepwise regression algorithm to build a predictive model of the outcome variable. For the personal finance site, simple Pearson correlation (*ρ*) was used to identify questions that correlate with the outcome.

In order to study the relationship between participation and contribution in greater detail after the studies were completed, we re-implemented each of the analyses using the same procedure. Before modeling the data, missing answers were filled with mean values from those users who had answered particular questions. To measure the predictive potential of each question we measured the change in model quality (as measured by fraction of variance explained by the model, *R* ^2^) that resulted from adding a particular question to a simple linear regression model. We refer to this change in model quality as Δ*R* ^2^. (Note that we also evaluated the adjusted *R* ^2^ statistic, but because our datasets are relatively large the use of adjusted *R* ^2^ did not substantially change the results.) In this stepwise regression the first variable added to the model was the one that most correlated (measured by *ρ* ^2^) with the outcome variable. The next variable to be added was the one that most correlated with the residual from the regression model. Since we were primarily interested to find the most important questions, and in order to avoid overfitting, we report only on the first five questions/variables to enter the model.

In order to gain deeper insight into the characteristics of high quality questions (see “Predicting High Quality Question Submissions”), we used a non-linear modeling method known as genetic programming (GP) [27]. Genetic programming is a nonlinear regression method that uses biologically-inspired, population-based metaheuristics that do not require the user to pre-specify terms for the regressed model. For our GP implementation, we used Nutonian’s Eureqa software [28][29]. Eureqa was chosen due to its track record as the preeminent nonlinear regression software package and ease of use. We employed most of the default options except that: we normalized and removed outliers as recommended by Eureqa, and we only allowed the use of the basic formula building blocks: constants, input variables, addition, subtraction, multiplication, and division. For each dataset we allowed Eureqa to run for 4×10^5^ function evaluations for each of the three datasets, which seemed sufficient to obtain a stable Pareto front of models that struck a balance between model accuracy and parsimony.

## Results

In this section, we analyze the rates of participation and the quality of contribution for each of the three crowdsourced surveys. We begin by reporting the descriptive statistics of each of the three studies in order to set the stage for further analysis. As users were asked to collectively discover predictive questions, we investigate the predictive power of the top questions in each study. We then characterize the rates of both response submissions and question submissions per user. Finally, we examine the quality of participant submissions and look into the relationship between the amount and quality of participation.

### Descriptive Statistics for the Three Studies

The three crowdsourcing studies that we examine in this paper focused on very different domains and were advertised to very different populations, and thus had different statistical properties. Table 1 reports descriptive statistics for the three crowdsourcing studies. Measures of overall participation include the number of users who participated, the number of questions injected, and the number of responses provided. The rate of participation is measured by the number of questions posted per user and the number of responses supplied per user. The percent of questions that are predictive of the response variable is determined by calculating the proportion of questions that correlated with it with a *p*-value of less than 0.05. The final four rows of Table 1 report the proportion of the different types of question collected during each experiment. Several similarities can be observed across the three studies. Most users posed, on average, less than one question. Between five and 13 percent of users posed at least one question. Between 10 and 22 percent of the user-posed questions were predictive of the outcome variable. Finally, given the choice of type of question to submit, the majority of participants in all three surveys chose to submit yes/no questions.

**Table 1:** Descriptive statistics of the three crowdsourced surveys.

	Childhood BMI	EnergyMinder	Personal Savings
Length of Deployment (in days)	12	179	23
Number of Active Users	533	606	2310
Number of Questions	59	591	289
Number of Responses	10,900	104,876	181,119
Mean Posted Questions per User	0.1061	0.7252	0.1251
Median Posted Questions per User	0	0	0
Max Posted Questions per User	9	318	35
% of Users to Post >0 Questions	6%	13%	5%
Mean Responses per User	19.60	119.58	78.41
Median Responses per User	15	65	20
Percent of Questions: Predictive	20.34%	10.06%	22.15%
Percent of Questions: Numerical	28.81%	21.84%	29.07%
Percent of Questions: Yes/No	54.24%	61.23%	70.24%
Percent of Questions: Likert Scale	16.95%	16.93%	N/A
Percent of Questions: User Defined	N/A	N/A	0.69%

One significant difference between the three studies was the length of deployment. Both the Childhood BMI and the Personal Savings studies were temporary in nature and were only active and advertised for two and four weeks respectively. Both studies were terminated after participation dropped to less than one new user per day. The EnergyMinder study is an ongoing study that has been active for about a year as of the writing of this paper. Another notable difference across all three studies was that the number of participants, questions submitted, and responses submitted all varied significantly. The most extreme observation of these differences is that a single user in the EnergyMinder study submitted 318 questions, whereas the maximum number of questions any single user submitted in the other two studies was 35 in the Personal Savings study and nine in the Childhood BMI study.

### Predictive Potential of Participant Questions

In each of the three crowdsourced surveys, the primary goal was to discover whether non-expert members of the public could generate questions that could be used to predict the response variable (Childhood BMI: adult body mass index; EnergyMinder: monthly home electricity usage in kilowatt-hours; Personal Savings: primary bank account balance). Tables 2, 3, and 4 report the most predictive questions for the three crowdsourcing studies, based on the Δ*R* ^2^ metric from stepwise linear regression (see Methods). In these tables, the Question I.D. field indicates the order in which the questions were submitted over the course of the study.

**Table 2:** Childhood BMI: Top 5 Questions by Rank in a Stepwise Linear Model.

Question I.D.	Question Text	Δ*R* ^2^
4	When you were a child: were your parents obese?	0.0376
53	When you were a child: did someone consistently pack a lunch for you to take to school?	0.0241
7	When you were a child: did your parents restrict your food intake?	0.0196
6	When you were a child: were you rewarded with food?	0.0114
59	When you were a child…did your parents talk about nutrition?	0.0114

**Table 3:** EnergyMinder: Top 5 Questions by Rank in a Stepwise Linear Model.

Question I.D.	Question Text	Δ*R* ^2^
4	How many loads of laundry do you do per week?	0.0657
34	How many months of the year do you use your dehumidifier?	0.0279
1	I generally use air conditioners on hot summer days	0.0220
42	Do you have a hot air heating system? (electric blower/fan)	0.0190
74	What’s the size of your home or apartment, in square feet?	0.0143

**Table 4:** Personal Savings: Top 5 Questions by Rank in a Stepwise Linear Model.

Question I.D.	Question Text	Δ*R* ^2^
413	Do you practice yoga?	1.05 × 10^−3^
412	Do you try to eat organic?	5.37 × 10^−5^
325	How many times per week do you exercise?	2.85 × 10^−5^
436	Do you set your thermostat above 70F (21C if not from US) in the winter?	1.96 × 10^−5^
405	What is the age of your savings account?	1.84 × 10^−5^

Tables 5, 6, and 7 report the questions that exhibited the highest absolute Pearson correlation coefficient ∣*ρ*∣ with the outcome variable. The Pearson correlation coefficients were calculated using complete observation pairs (missing responses were not considered) and only questions with at least 50 complete observations were considered.

**Table 5:** Childhood BMI: Top 5 Correlated Questions by Pearson Correlation Coefficient with the Response Variable.

Question I.D.	Question Text	*ρ*
53	When you were a child, did someone consistently pack a lunch for you to take to school?	-0.341
59	When you were a child…did your parents talk about nutrition?	-0.301
34	When you were a child…did your family primarily prepare meals using fresh ingredients?	-0.289
39	When you were a child, was the food used as a punishment in any ways?	0.231
4	When you were a child, were your parents obese?	0.230

**Table 6:** EnergyMinder: Top 5 Correlated Questions by Pearson Correlation Coefficient with the Response Variable.

Question I.D.	Question Text	*ρ*
4	How many loads of laundry do you do per week?	0.334
393	I generally turn my heat on before October.	0.331
34	How many months of the year do you use your dehumidifier?	0.313
74	What’s the size of your home or apartment, in square feet?	0.310
258	How many times do you use a towel before throwing it into the laundry?	-0.309

**Table 7:** Personal Savings: Top 5 Correlated Questions by Pearson Correlation Coefficient with the Response Variable.

Question I.D.	Question Text	*ρ*
201	How much money do you have in your emergency fund?	0.621
400	How many months of living expenses do you have saved?	0.559
224	How much money do you currently have invested in bonds?	0.360
209	What is your net worth?	0.345
422	How much money do you put into a retirement account each year?	0.327

Considering the Pearson correlation of each question against the response variable as reported in Table 1, we found that a substantial fraction of the questions were predictive (*p*-values of <0.05 for the hypothesis that the correlation is significantly different from zero) in each study (Childhood BMI: 20.34%; EnergyMinder: 10.06%; Personal Savings: 22.15%). This suggests that participants have a good sense of what types of questions are appropriate when trying to predict the response variable. As in a previous study [26], we found that while many questions are ones that an expert would be expected to generate, some questions were surprising. One such question was “Do you practice yoga?” in the Personal Savings survey. Although physical fitness and physical recreation are known to correlate with socioeconomic status [30], a link between yoga and SES could not be found in the literature. This observation suggests that instead of asking many finance-specific questions, perhaps the crowd could discover a small number of lifestyle questions that are mutually orthogonal yet collectively achieve the same amount of predictive power. We believe that these non-traditional yet predictive questions highlight the potential of crowdsourced surveys.

### Honesty of Participant Question Responses

Determining the quality of user-generated survey questions requires collecting many accurate responses for each of them. When conducting a survey on the internet where participation is anonymous, the honesty of responses, or lack thereof, is a common concern [31]. However, it is difficult to determine which responses are truthful and which are not. For questions with built-in valid response ranges, it is possible to detect inaccurate responses. An example question is, “What is your age?” The oldest known person is Misao Okawa of Osaka, Japan, who is 116 years old. If any participants responded that they were more than 116 or less than zero years old, the falsity of their response is clear. Each dataset included a sample of questions with valid ranges, proving a useful proxy measure of veracity in crowdsourced surveys.

In the Childhood BMI dataset, of the 59 questions posed, eight contained built-in valid ranges of response. Participants provided 943 responses to those questions and of those, six responses lay outside of the valid ranges (0.64%). In the EnergyMinder dataset, of the 591 questions posed, 42 contained built-in valid ranges of response. Participants provided 7,364 responses to those questions and of those, 13 responses lay outside of the valid ranges (0.18%). In the Personal Savings dataset, of the 289 questions posed, 18 contained built-in valid ranges of response. Participants provided 11,562 responses to those questions and of those, three responses lay outside of the valid ranges (0.03%). These results suggest that the majority of participants answered truthfully a majority of the time, at least to questions with built-in ranges.

### Participation Rates of Question and Response Submissions

In addition to examining the quality of question and response submissions, we also examined the rates of question and response submissions. Based on prior research, we expected to find that the amount of participation per user would follow a power-law distribution with a small number of users submitting many responses and questions and the majority of users submitting a small number of responses and questions.

**Fig 2:**
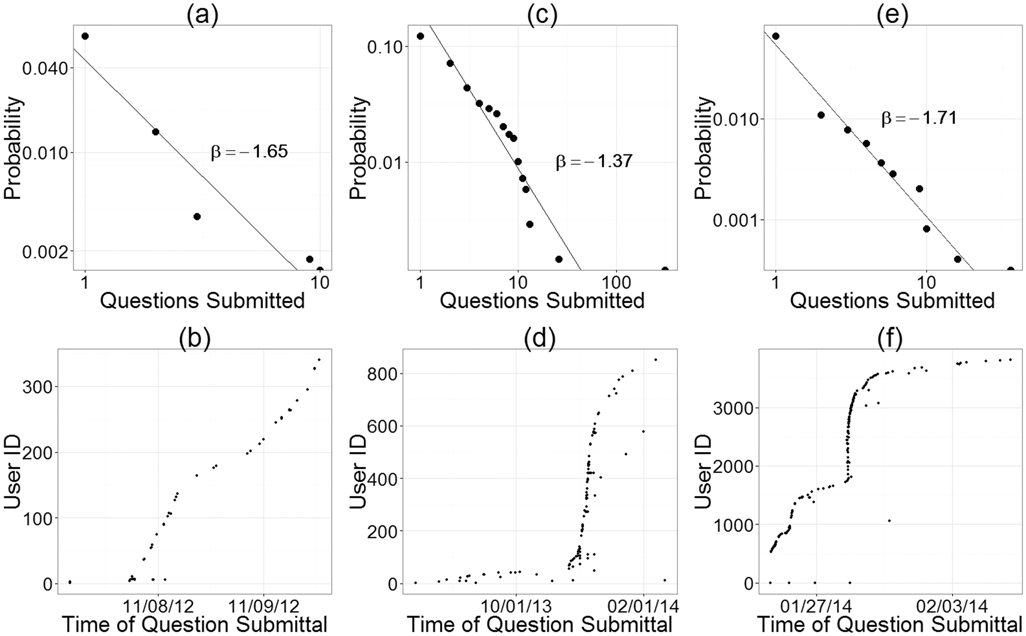
Number of Questions Submitted. Plots (a) and (b) use the Childhood BMI data, plots (c) and (d) use the EnergyMinder data, and plots (e) and (f) use the Personal Savings data. Plots (a), (c), and (e) display probability distributions for the number of questions submitted per user using *log*_10_ axes, along with power-law fit lines. Plots (b), (d), and (f) display the times at which users submitted questions. User ID was assigned based on time of first arrival to the surveys.

Fig. 2 reports the distributions of questions submitted per user in each of the three studies as well as when those questions were submitted over the course of the study. Fig. 2a, c, and e indicate that the number of questions submitted follows a heavy tailed distribution. There were insufficient data to confirm statistically that these distributions could be fit with a power law, but they were similar to such a distribution. This means that in each of the three studies, a small percentage of participants submitted the majority of the questions, while most participants submitted no questions.

The rate of question submissions also varied greatly over time. Fig. 2d and 2f, in particular, indicate sharp spikes in user traffic during which the number of questions submitted rose significantly over a short period of time. These sharp rises correspond to sharp rises in overall participation. During the EnergyMinder survey, the sharp upturn in questions submitted occured during the second round of a directed recruitment campaign. During the Personal Savings survey, the sharpest upturn in questions submitted occurred due to the mention of the experiment on a popular finance blog.

**Fig 3:**
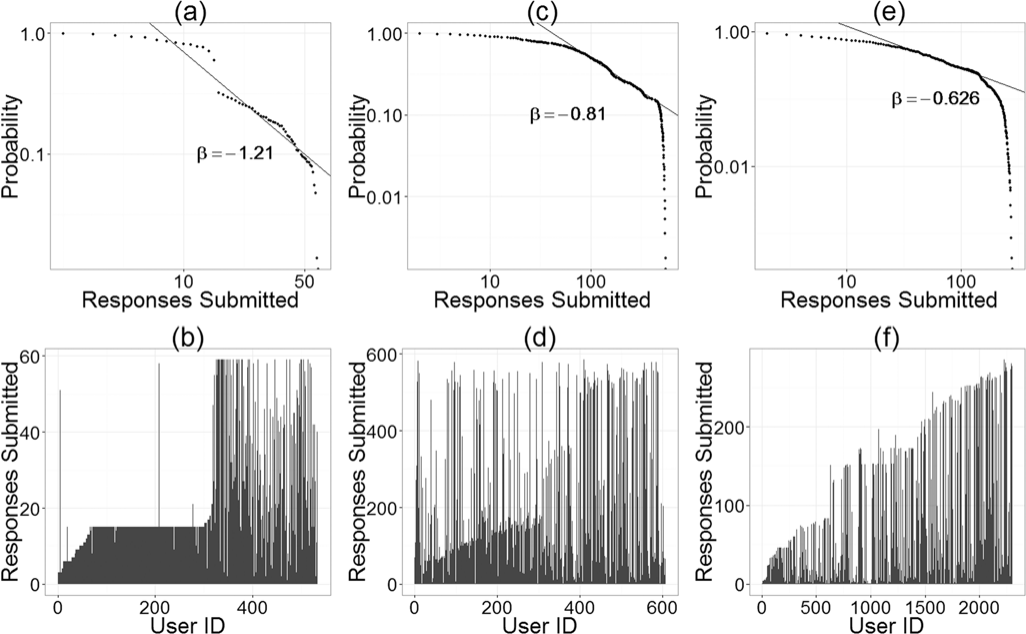
Number of Responses Submitted. Plots (a) and (b) use the Childhood BMI data, plots (c) and (d) use the EnergyMinder data, and plots (e) and (f) use the Personal Savings data. Plots (a), (c), and (e), display (complementary) cumulative probability distributions for the number of responses submitted per user, along with power-law fit lines. Plots (b), (d), and (f), display the number of responses submitted for each participant sorted by User ID (which were assigned based on time of first arrival).

Fig. 3 reports the distribution of responses submitted per user in each of the three studies. The plateaus that can be observed in Fig. 3b and 3f result from two causes. Before being released into the survey, user-generated questions are filtered by a human moderator. Intermittent moderation led to a backlog of questions which created time periods during which only a fixed number of questions are available. Alternatively, there were intermittent periods during which no new questions were posed by the crowd.

Fig. 3a, c, e do not indicate that there is a heavy-tailed distribution in the number of responses submitted per user: there is a fairly uniform spread between users who only submitted one response up to users who attempted to respond to every question on the surveys. Part of the explaination for this is the mechanism by which the surveys grew in size. The first participants to come to the site only saw the few seed questions that were provided at the start of each study (Childhood BMI: 3; EnergyMinder: 6; Personal Savings: 1). Participants that visited the surveys later saw all of the participant-provided questions that had been accepted up until that point and therefore had the opportunity to submit a greater number of responses than earlier participants. Participants could choose to re-visit the site at a later time and answer any questions that had been added to the survey since they had last been to the survey. Fig. 3d shows that this did occur with the EnergyMinder survey. User IDs were assigned based on the time that a participant first visited the site. As shown in Fig. 3d, there are many participants with relatively low IDs who answered a large percentage of the total questions. These participants visited the survey early but returned at a later date and answered more questions. In contrast, Fig. 3b and 3f suggest that many early participants did not return.

### Participation versus Contribution

We were next interested to determine if there was a relationship between the amount of participation per user and their contribution (quality of participation). If a positive correlation is discovered between participation and contribution, one might hypothesize that experts in the particular domain of interest were more motivated to provide questions than laypersons. Alternatively, a negative correlation could be the result of users attempting to vandalize or deface the website by contributing larger amounts of poor or random content, compared to engaged and invested users.

**Fig 4:**
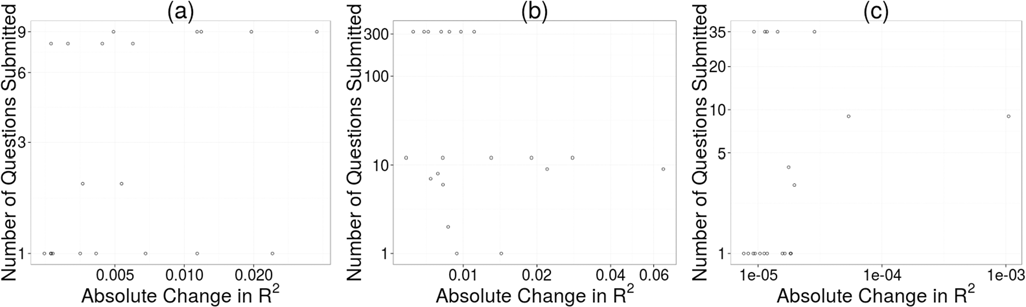
Participation versus Contribution: number of questions submitted per user plotted against question contribution. Plot (a) uses the Childhood BMI data, plot (b) uses the EnergyMinder data, and plot (c) uses the Personal Savings data.

Fig. 4 reports the relationship between participation and contribution of question submissions for the three studies. For these figures we collected the first 20 questions found by a stepwise linear model (using Pearson correlation against the residuals as the decision mechanism) from each dataset and found no significant correlations between participation and contribution across all three studies (Pearson correlation, *ρ* = 0.32, *p* = 0.16; *ρ* = −0.29, *p* = 0.21; *ρ* = 0.24, *p* = 0.31). We also calculated Pearson correlation coefficients using up to 50 of the most predictive questions to see if including more questions would produce a significant correlation. For the Childhood BMI dataset, we found that of these 31 correlations (top 20 questions to top 50 questions), 8 were significantly correlated with p-values of less than 0.05. When there was a significant correlation, the Pearson correlation coefficient was always positive (ranging from 0.30 to 0.48). For the EnergyMinder dataset, we found that 13 of the correlations were significant, and when they were significant, the Pearson coefficient was always negative (ranging from −0.34 to −0.42). Finally, for the Personal Savings dataset, we found that 12 of the correlations were significant, and when they were significant, the Pearson coefficient was always positive (ranging from 0.31 to 0.38). In sum, we do not find evidence of a relationship between participation and contribution of questions submitted: the questions submitted by lightly participating users were just as likely to be predictive as questions submitted by heavily participating users.

**Fig 5:**
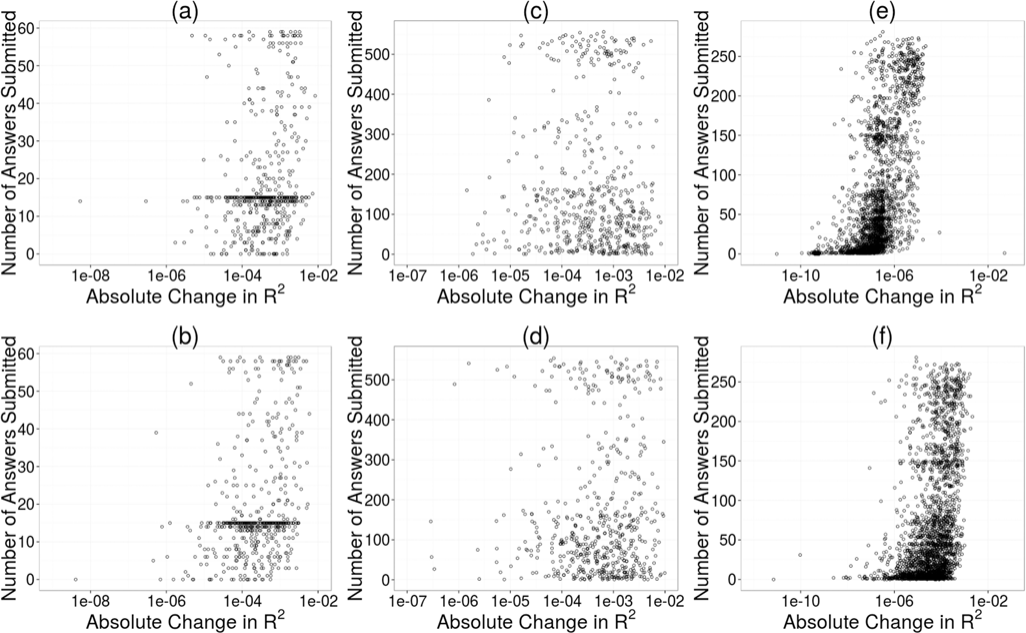
Participation versus Contribution: number of responses submitted per user plotted against response contribution per user. Plots (a) and (b) use the Childhood BMI data, plots (c) and (d) use the EnergyMinder data, and plots (e) and (f) use the Personal Savings data. In plots (a), (c), and (e), real survey data is used. In plots (b), (d), and (f), synthetic data is used.

Fig. 5a, c, e reports the relationship between participation and contribution of response submissions across the three studies. The horizontal axes correspond to the absolute value of the change in *R* ^2^ in a linear model when a participant’s responses were removed from the data. Significant correlation between participation and contribution of responses was found for the Childhood BMI dataset and the Personal Savings dataset (Pearson correlation, *ρ* = 0.18, *p* < 0.001; *ρ* = 0.57, *p* < 0.001). This means that the more responses that a user submitted, the more their responses affected a linear model. There are two possible explanations for this result. Either users who answered more questions were somehow more valued by a linear model (possibly due to uniqueness of responses), or simply answering more questions (and therefore having fewer mean substituted values) made the user more valuable to the model.

To disambiguate between these two hypotheses, synthetic datasets were generated that were similar to the three crowdsourced datasets. If synthetic data results in the same significant correlations between quantity and quality of responses, then the existence of the correlations must be a result of the number of responses provided, regardless of what those responses actually are. These synthetic datasets had missing values in the same locations as in the actual datasets, and the synthetic responses to each question were generated using a Gaussian distribution to conform to the same mean and standard deviation as the actual datasets. When we ran our analysis on the synthetic data, we obtained similar results as with the actual data (Fig. 5b, d, f). We found significant correlations between contribution of responses and number of responses submitted for both the synthetic Childhood BMI data as well as the synthetic Personal Savings data (Pearson correlation, *ρ* = 0.25, *p* < 0.001; *ρ* = 0.38, *p* < 0.001). This suggests that there is no significant correlation between the synthetic number of responses submitted and the quality of those responses, and that the correlation between quantity and quality of responses in the two crowdsourced datasets is spurious, caused by missing data and mean substitution.

### Investigating the Provenance of High-Quality Questions

Finally, we attempted to gain a deeper understanding of the provenance of high-quality question submissions. In the previous section we found that participants that pose a greater number of questions do not necessarily pose better questions. This raised the question of whether there were any other characteristics, or combinations of characteristics, that predict the quality of question submissions. To investigate this we measured the following features for each question across the three studies:

‘**c**’—The contribution of this question when predicting the outcome variable (Childhood BMI: adult body mass index; EnergyMinder: monthly home electricity usage in kilowatt-hours; Personal Savings: primary bank account balance). This was calculated using the square of the Pearson correlation coefficient (*ρ* ^2^) for each question against the outcome variable. This feature was used as the dependent variable in the following analyses.
‘**tos**’—The number of seconds that elapsed between the commencement of the experiment and the submission of the question.
‘**q_resp**’—The total number of responses submitted by all users for this question.
‘**qs_sub**’—The total number of questions submitted by the user who submitted this question.
‘**rs_sub**’—The total number of responses to all questions submitted by the user who submitted this question.
‘**dep_dev**’—The *z*-score (standard deviations above or below the mean) representation of the outcome value for the user who submitted this question.
‘**tbq**’—The number of seconds that have elapsed between the arrival of the user who submitted this question to the survey and the time of this question’s submission.
‘**rbq**’—The total number of question responses submitted by the user who submitted this question before submitting this question.
‘**qbq**’—The total number of questions submitted by the user who submitted this question before submitting this question.

We hypothesize that these question characteristics may partially explain why some questions were predictive, and others were not. Our reasoning for formulating the ‘tos’,‘qs_sub’, ‘rs_sub’, ‘tbq’, ‘rbq’, and ‘qbq’ variables was that the more a user was involved with the survey and was exposed to others’ questions, the more likely (or unlikely) they might be to submit better questions. The ‘q_resp’ variable was introduced to control for the varying number of responses each question received. Finally, the ‘dep_dev’ variable was included because we surmised that users with extreme values of the response variable may have more insight—or be more motivated—than the average participant. For example, a participant in the EnergyMinder survey who had a very high electricity bill might be more invested in understanding their energy usage than a participant with a monthly electricity bill closer to the mean value.

We began by regressing the eight independent variables above in a single model against the ‘c’ variable in addition to calculating the Pearson correlation coefficients (Table 8). These results report that for the Childhood BMI and Personal Savings datasets, none of the eight variables are significant linear predictors of question contribution. For the EnergyMinder dataset, the variables ‘tos’, ‘q_resp’, ‘rs_sub’, dep_dev, and ‘rbq’ are all significant at a *p* = 0.05 level. These results suggest that these variables may be able to provide insight into where high quality questions come from. To take the analysis further, we chose to examine non-linear combinations of these variables using a nonlinear regression method (see Methods).

**Table 8:** Predicting Question Quality.

	Chilidhood BMI		EnergyMinder		Personal Savings	
Variable	Reg. Coef.	*ρ*	Reg. Coef.	*ρ*	Reg. Coef.	*ρ*
tos	2.91 × 10^−2^	0.209	−2.14 × 10^−2^*	−0.214***	8.93 × 10^−3^	−3.77 × 10^−2^
q_resp	−2.02 × 10^−5^	−0.159	−6.34 × 10^−5^	0.125*	2.38 × 10^−5^	4.64 × 10^−2^
qs_sub	2.45 × 10^−3^	0.068	4.26 × 10^−4^	0.018	−1.01 × 10^−4^	−3.70 × 10^−2^
rs_sub	−1.58 × 10^−4^	0.103	−5.83 × 10^−6^	−0.130*	3.02 × 10^−5^	−2.41 × 10^−2^
dep_dev	5.44 × 10^−4^	0.062	3.50 × 10^−3^	0.144*	−5.85	−3.67 × 10^−2^
tbq	2.70 × 10^−6^	0.121	−5.99 × 10^−10^	−0.052	−1.83 × 10^−8^	−5.72 × 10^−2^
rbq	−6.35 × 10^−4^	0.089	−2.13 × 10^−5^*	−0.189**	3.40 × 10^−5^	−6.58 × 10^−3^
qbq	−3.53 × 10^−3^	0.036	−2.99 × 10^−4^	−0.070	−4.93 × 10^−5^	−3.44 × 10^−2^

Next, we generated models to predict the contribution of questions to model quality (‘c’) using Nutonian’s Eureqa genetic programming software [28][29]. Tables 9, 10, and 11 report the linear and non-linear models that were obtained. One large difference between the three tables is the number and size of models in Table 9 compared with the number and size of models in Tables 10, and 11. We believe the reason for this difference is due to the difference in the number of questions in the three datasets. The Childhood BMI study terminated with 59 questions, while the EnergyMinder and Personal Savings studies terminated with 591 and 289 questions respectively. Due to the small number of questions in the Childhood BMI dataset, Eureqa most likely over-fit the data with many of the larger models. Due to the larger sizes of the models there are many possible variations that show up in the suite of models. Similarly, Eureqa appears to have over-fit the Personal Savings data. As reported in Table 11, the three largest models (sizes 20, 18, and 16) all have very low *R* ^2^ values, indicating that these models are not particularly insightful.

**Table 9:** Childhood BMI: Predicting Question Quality using Genetic Programming.

Size of Model	*R* ^2^	Model
48	0.214	*c* = (1.60 × 10^−3^ * *qs*_*sub* + 2.86 × 10^−4^ * *dep*_*dev* * *qs*_*sub* ^3^ + 1.84 × 10^−3^ * *qs*_*sub* * *dep*_*dev* * *qbq* ^2^ − 2.87 × 10^−4^ * *qbq* − 2.87 × 10^−4^ * *dep*_*dev* ^2^ − 1.75 × 10^−3^ * *dep*_*dev* * *qbq* * *qs*_*sub* ^2^)/(1.04 − *tos*)
44	0.210	*c* = (1.60 × 10^−3^ * *qs*_*sub* + 2.85 × 10^−4^ * *dep*_*dev* * *qs*_*sub* ^3^ + 1.83 × 10^−3^ * *qs*_*sub* * *dep*_*dev* * *qbq* ^2^ − 3.14 × 10^−4^ * *dep*_*dev* ^2^−1.77 × 10^−3^ * *dep*_*dev* * *qbq* * *qs*_*sub* ^2^)/(1.042 − *tos*)
42	0.205	*c* = (1.75 × 10^−3^ * *qs*_*sub* + 2.77 × 10^−4^ * *dep*_*dev* * *qs*_*sub* ^3^ + 1.87 × 10^−3^ * *qs*_*sub* * *dep*_*dev* * *qbq* ^2^ − 5.55 × 10^−4^ * *qbq* − 1.76 × 10^−3^ * *dep*_*dev* * *qbq* * *qs*_*sub* ^2^)/(1.046 − *tos*)
38	0.202	*c* = (1.46 × 10^−3^ * *qs*_*sub* + 2.49 × 10^−4^ * *dep*_*dev* * *qs*_*sub* ^3^ + 1.69 × 10^−3^ * *qs*_*sub* * *dep*_*dev* * *qbq* ^2^ − 1.60 × 10^−3^ * *dep*_*dev* * *qbq* * *qs*_*sub* ^2^)/(1.04 − *tos*)
32	0.151	*c* = (1.72 × 10^−3^ * *qs*_*sub* + 3.68 × 10^−3^ * *dep*_*dev* * *qbq* ^2^ + 2.60 × 10^−4^ * *dep*_*dev* * *qs*_*sub* ^2^ − 3.19 × 10^−3^ * *qs*_*sub* * *dep*_*dev* * *qbq*)/(1.05 − *tos*)
30	0.158	*c* = (1.54 × 10^−3^ * *qs*_*sub* + 8.32 × 10^−4^ * *qs*_*sub* * *dep*_*dev* + 6.04 × 10^−3^ * *dep*_*dev* * *qbq* ^2^ − 4.60 × 10^−3^ * *qs*_*sub* * *dep*_*dev* * *qbq*)/(1.04 − *tos*)
28	0.159	*c* = (1.54 × 10^−3^ * *qs*_*sub* + 1.04 × 10^−3^ * *dep*_*dev* + 5.83 × 10^−3^ * *dep*_*dev* * *qbq* ^2^ − 4.32 × 10^−3^ * *qs*_*sub* * *dep*_*dev* * *qbq*)/(1.04 − *tos*)
24	0.125	*c* = (2.17 × 10^−3^ * *qs*_*sub* + 4.56 × 10^−4^ * *dep*_*dev* − 9.88 × 10^−7^ − 3.74 × 10^−4^ * *qbq* − 2.37 × 10^−3^ * *dep*_*dev* * *qbq*)/(1.06 − *tos*)
22	0.125	*c* = (2.17 × 10^−3^ * *qs*_*sub* + 4.55 × 10^−4^ * *dep*_*dev* − 3.74 × 10^−4^ * *qbq* − 2.37 × 10^−3^ * *dep*_*dev* * *qbq*)/(1.06 − *tos*)
20	0.119	*c* = (2.00 × 10^−3^ * *qs*_*sub* + 2.80 × 10^−4^ * *qs*_*sub* * *dep*_*dev* − 2.49 × 10^−3^ * *dep*_*dev* * *qbq*)/(1.05 − *tos*)
18	0.122	*c* = (2.03 × 10^−3^ * *qs*_*sub* + 5.28 × 10^−4^ * *dep*_*dev* − 2.49 × 10^−3^ * *dep*_*dev* * *qbq*)/(1.05 − *tos*)
16	0.121	*c* = (7.66 × 10^−8^ + 2.56 × 10^−3^ * *qs*_*sub* − 5.80 × 10^−3^ * *tos* * *qbq*)/(1.07 − *tos*)
14	0.121	*c* = (2.56 × 10^−3^ * *qs*_*sub* − 5.80 × 10^−3^ * *tos* * *qbq*)/(1.07 − *tos*)
12	0.109	*c* = (2.26 × 10^−3^ * *qs*_*sub* − 1.37 × 10^−3^ * *qbq*)/(1.06 − *tos*)
10	0.081	*c* = 2.12 × 10^−3^ * *qs*_*sub*/(1.05 − *tos*) − 2.45 × 10^−4^
8	0.081	*c* = 2.09 × 10^−3^ * *qs*_*sub*/(1.05 − *tos*)
7	0.055	*c* = 1.92 × 10^−2^ * *qs*_*sub* * *tos* ^2^
5	0.017	*c* = 1.07 × 10^−2^ * *tos* * *qs*_*sub*
3	0.003	*c* = 2.57 × 10^−3^ * *qs*_*sub*
1	0	*c* = 8.27 × 10^−3^

**Table 10:** EnergyMinder: Predicting Question Quality using Genetic Programming.

Size of Model	*R* ^2^	Model
24	0.226	*c* = *qs*_*sub*/(205.41 * *qbq* + 55.47 * *qs*_*sub* + *rbq* ^2^ − *qbq* * *qs*_*sub* ^2^ − 13.94 * *rbq*)
20	0.199	*c* = *qs*_*sub*/(51.69 * *qs*_*sub* + *rbq* ^2^ + *tos* * *q*_*resp* * *qbq* − 13.52 * *rbq*)
18	0.194	*c* = *qs*_*sub*/(51.69 * *qs*_*sub* + 49.61 * *qbq* + *rbq* ^2^ − 13.52 * *rbq*)
16	0.177	*c* = *qs*_*sub*/(75.39 * *qs*_*sub* + *rbq* ^2^ − 23.26 − 13.57 * *rbq*)
14	0.187	*c* = *qs*_*sub*/(52.56 * *qs*_*sub* + *rbq* ^2^ − 13.62 * *rbq*)
10	0.108	*c* = 0.22/(19.23 + *rbq* + 23.88 * *dep*_*dev*)
8	0.061	*c* = 6.24 × 10^−2^/(43.21 + 39.40 * *dep*_*dev*)
6	0.060	*c* = 1.61 × 10^−3^/(1.10 + *dep*_*dev*)
5	0.063	*c* = 6.96 × 10^−3^ − 8.05 × 10^−3^ * *tos*
3	0.022	*c* = 1.18 × 10^−5^ * *q*_*resp*
1	0	*c* = 2.27 × 10^−3^

**Table 11:** Personal Savings: Predicting Question Quality using Genetic Programming.

Size of Model	*R* ^2^	Model
20	0.028	*c* = 5.27 × 10^−4^ + 5.22 × 10^−3^/(969.04 + 1.05 × 10^−2^ * *q*_*resp* ^2^ − 0.27 * *qs*_*sub* − 6.38 * *q*_*resp*) − 1.14 × 10^−7^ * *tbq*
18	0.023	*c* = 1.64 × 10^−4^ + 5.22 × 10^−3^/(969.03 + 1.05 × 10^−2^ * *q*_*resp* ^2^ − 0.27 * *qs*_*sub* − 6.38 * *q*_*resp*)
16	0.016	*c* = 1.76 × 10^−4^ + 3.24 × 10^−3^/(968.18 + 1.05 × 10^−2^ * *q*_*resp* ^2^ − 6.38 * *q*_*resp*)
14	0.071	*c* = 1.08 × 10^−3^ + (2.74 × 10^−5^ * *q*_*resp* − 1.73 × 10^−2^)/(*rs*_*sub* − 132.04) − 1.14 × 10^−7^ * *tbq* − 5.14 × 10^−5^ * *qs*_*sub*
12	0.062	*c* = 8.18 × 10^−4^ + (2.78 × 10^−5^ * *q*_*resp* − 1.76 × 10^−2^)/(*rs*_*sub* − 132.04) − 4.78 × 10^−5^ * *qs*_*sub*
10	0.053	*c* = 7.41 × 10^−4^ + (2.72 × 10^−5^ * *q*_*resp* − 1.72 × 10^−2^)/(*rs*_*sub* − 132.04) − 1.14 × 10^−7^ * *tbq*
8	0.031	*c* = 2.33 × 10^−4^ + 1.99 × 10^−6^/(2.44 + 1.40 × 10^−4^ * *rs*_*sub* ^2^ − 3.69 × 10^−2^ * *rs*_*sub*)
7	0.037	*c* = 9.22 × 10^−4^ − 1.14 × 10^−7^ * *tbq* − 5.06 × 10^−5^ * *qs*_*sub*
6	0.023	*c* = 5.55 × 10^−4^ + (3.61 × 10^−5^ * *qs*_*sub* − 1.25 × 10^−4^)/(*tos* − 0.68)
5	0.022	*c* = 6.58 × 10^−4^ − 4.39 × 10^−5^ * *qs*_*sub*
3	0.012	*c* = 6.24 × 10^−4^ − 1.14 × 10^−7^ * *tbq*
1	0	*c* = 3.00 × 10^−4^

However, we did eventually find useful insight from the EnergyMinder dataset. Initially, we did not find consistent trends in the modeling results (and very low *R* ^2^ values). We hypothesized that these poor results were due to the one participant who submitted 318 of the 591 questions to the survey. Due to one individual submitting so many questions, many of the eight variables described above had nearly identical values for all 318 observations in our dataset. For this reason, this participant was removed from the dataset and only the remaining 273 questions were considered.

**Fig 6:**
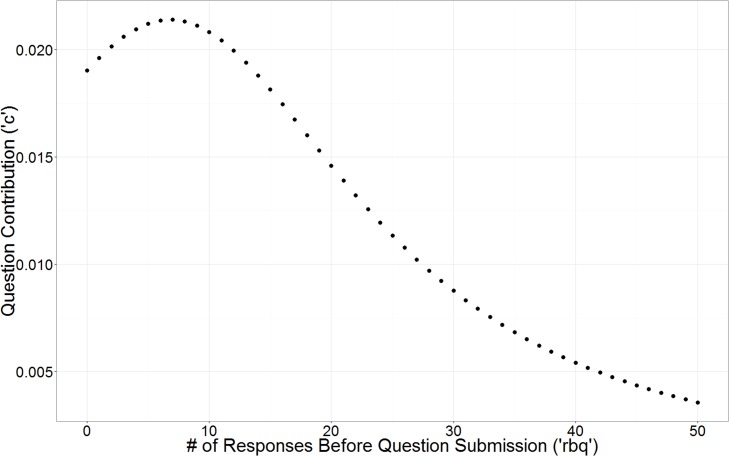
Question contribution after submitting responses in the EnergyMinder survey. The model of size 14 from Table 10 is used to investigate the relationship between question contribution and the number of responses submitted before the user submitted their question. The variable ‘qs_sub’ is kept constant at the mean value of 7.98.

Table 10 reports the models found by Eureqa using this reduced EnergyMinder dataset. The variables ‘rbq’ and ‘qpq’ appear in many of the models, and always in the denominator. Along with ‘tos’ and ‘tbq’, these four variables are proxies for the amount of activity that occurs before the question is submitted. While variable ‘tbq’ does not appear in any of the models, the other three variables all appear in models in an inverse context. This suggests that questions submitted later in the study were not as predictive of the outcome variable. However this is just a generalization that does not always hold true as we see when looking at the placement of variable ‘rbq’ in the five largest models. In these five models, ‘rbq’ appears twice in the denominator: once as a squared term and once with a negative coefficient. This pattern of usage of the variable ‘rbq’ begins in the model of size 14 and, along with the usage of ‘qs_sub’, results in a substantial increase in *R* ^2^ over the model of size 10. A roughly linear positive relationship exists between the variable ‘qs_sub’ and ‘c’. For ‘rbq’, the net effect of these two appearences in the model of size 14 impacts ‘c’ in a positive way when the user had submitted fewer than eight responses before posing their question and negatively when the user had submitted more than eight responses before posing their question (Fig. 6). We found that the average number of responses submitted before question submission was 174, so more often than not, ‘rbq’ is used in these models to reduce the value of ‘c’, yet it is useful to know that at low values of ‘rbq’, it actually increases the value of ‘c’ in these models. This suggests that questions submitted late in the study, after participants had already contributed a great deal, were of relatively low quality. However, taking the time to submit a few responses before submitting a question proved beneficial to question quality. We conclude from this that there appears to be a “sweet spot” when participating in a crowdsourced survey: it is best to submit a few responses before submitting a question, but not too many.

## Discussion and Conclusions

Here we have characterized how participants interacted with three crowdsourced surveys in a variety of ways. In each of the three studies we found that participants generated a substantial number of questions that predict the response variable. We also found that participants did not submit obviously false responses which suggests that a majority of responses are honest. The distributions of question submissions per user followed heavy-tailed distributions as seen in many other crowdsourcing studies, while the distributions of response submissions per user lay closer to a uniform distribution. We found that higher rates of submission did not correlate with quality of contribution for both response submissions and question submissions. However, we did find that participants may submit higher quality questions after participants answer a few, but not too many, questions. After uses answered more than about 8 questions, the ultimate predictive power of the questions they posed decreased.

The very different distributions between question and response submissions show that even slight changes in the difficulty of tasks can have significant impacts on rates of participation. When designing these surveys, we strove to make question submission as easy as possible, yet clearly, posing a question is still much more difficult for the average user than simply responding to questions. It is possible that by using the word “survey”, participants were primed from the outset that their only task was to respond to questions. Reducing the barriers to question submission—and better incentivizing it—is of the highest priority for future work. One potentially fruitful approach would be to add game-like elements to these sites, building on the growing literature on gamification [32].

The lack of correlation between participation and contribution falsifies the hypothesis that a higher level of participation is indicative of interest in and knowledge of the subject area. The fact that this relationship does not exist has broad implications for future crowdsourcing work. Large numbers of casual users who participate very little are as valuable as a smaller number of users who participate a great deal. This suggests that any combination of attracting new users and retaining existing users is a valid strategy for collecting high-quality contribution and the resulting mixed strategy should be based on relative costs. With our crowdsourced surveys there is often less effort involved in attracting new users than retaining existing users. Our optimal strategy would therefore consist of more effort being expended in attracting new users than retaining existing users. In other crowdsourcing situations where attracting new users is relatively difficult, more effort should go into retaining users.

Further analysis did however suggest that participants submit higher quality questions before participating too much. This falsifies the hypothesis that users would be motivated by the questions already included in the survey to generate better questions. This result deserves more experimentation to verify, but could indicate that making an effort to encourage participants to submit questions when they first arrive at the survey is a worthwhile endeavor. Two hypotheses may explain this observation. It is possible that users are more likely to think more creatively when first arriving. Alternatively, it is possible that there are a limited number of good predictors for users to submit as questions, and once those are submitted, subsequent users are unable to formulate questions that are as predictive. Both hypotheses seem plausible and require further experimentation to verify.

We believe that, while preliminary, these results offer considerable insight to future crowdsourcing work. Understanding what characteristics enable crowdsourcing participants to generate high quality content is the first step in being able to empower all users to generate better content. For example, the finding that the best questions were submitted after some, but not too much, activity on the sites suggests that future crowdsourced surveys may benefit from prominently displaying a prompt to encourage question submission only after the user has submitted a certain number of responses and before they submit too many. (In the EnergyMinder case the optimal number of questions answered was eight.) In addition, the finding that some users submit many more questions than others without a measurable increase in quality suggests that it may be beneficial to limit question submission once a user has already participated a great deal.

## Ethics Statement

We obtained permission to conduct human subjects research from the Institutional Review Boards at the University of Vermont. Our IRB proposal was entitled “Wikinnovation: Automating and Democratizing Online Survey Construction.” Participants granted written consent by clicking on a button to proceed to the online survey after watching a video outlining the purpose of the crowdsourced surveys.

## Data Access

The data obtained from our three crowdsourced surveys is freely accessible under the MIT license from https://github.com/BobFromUVM/crowdsurvey2014. All images used in this paper are also accessible from the above URL under the MIT license.

## Supporting Information

S1 File Screen captures for the three crowdsourcing websites. (PDF)
