# Supplementary material for: Participation and Contribution in Crowdsourced Surveys
Source: PLoS One. 2015 Apr 2;10(4):e0120521. doi: 10.1371/journal.pone.0120521 (PMC4383627; doi:10.1371/journal.pone.0120521)
Supplement: S1 File — (PDF) [file pone.0120521.s001.pdf]

Supporting Information for: Participation and Contribution in Crowdsourced Surveys

Robert Swain, Alex Berger, Josh Bongard, and Paul Hines

This supporting information file provides screen captures showing various pages from each of the three crowdsourcing sites: Childhood BMI, EnergyMinder, and Personal Finance.

Childhood BMI

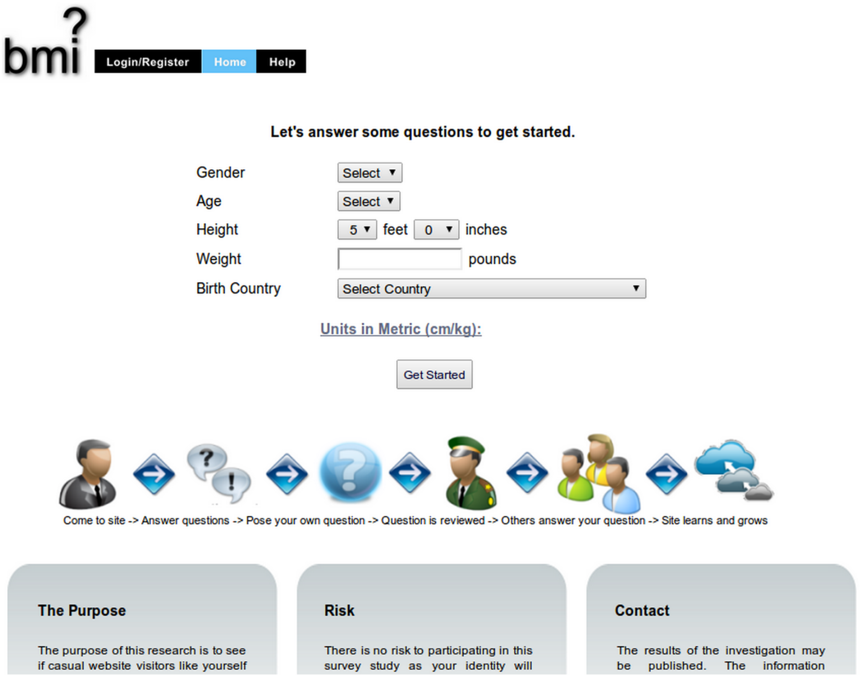

Figure A. Landing Page for the Childhood BMI site.

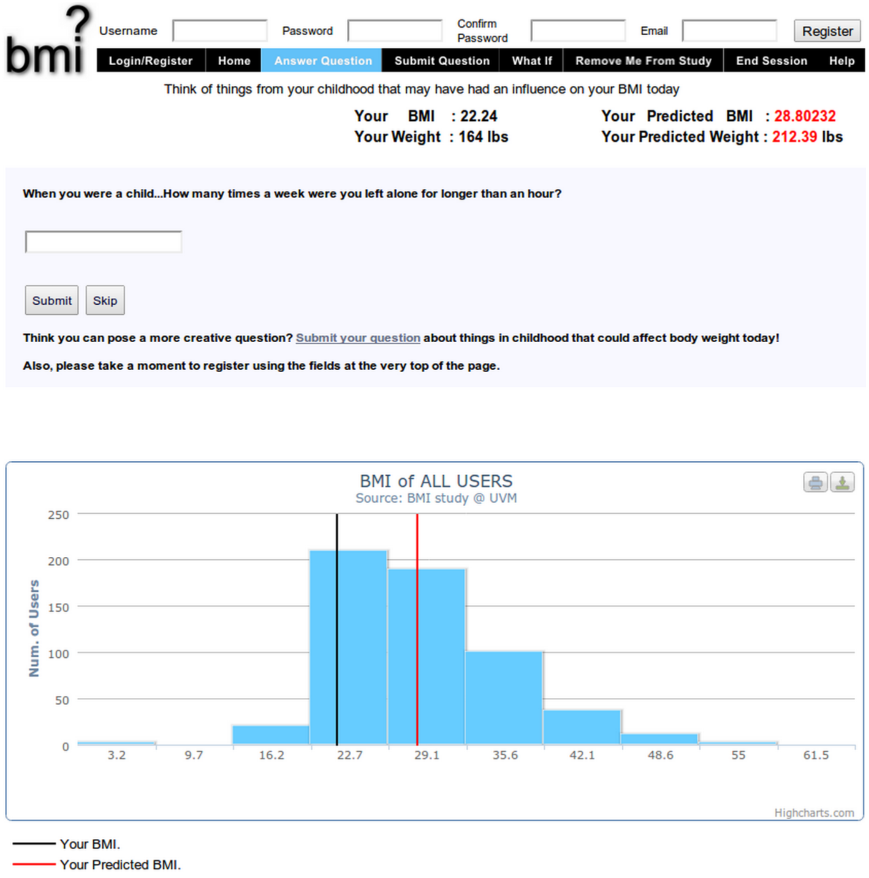

Figure B. Primary Survey Page for the Childhood BMI site.

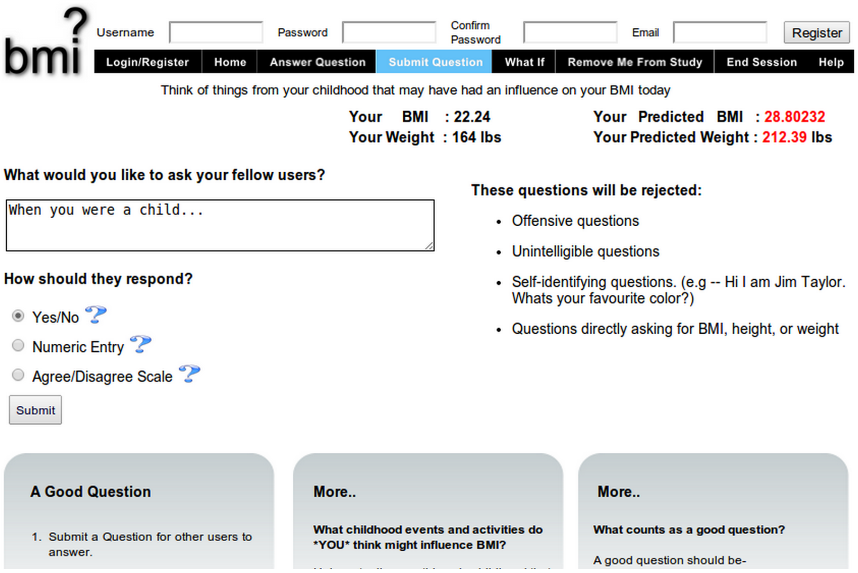

Figure C. Question Submittal Page for the Childhood BMI site.

EnergyMinder

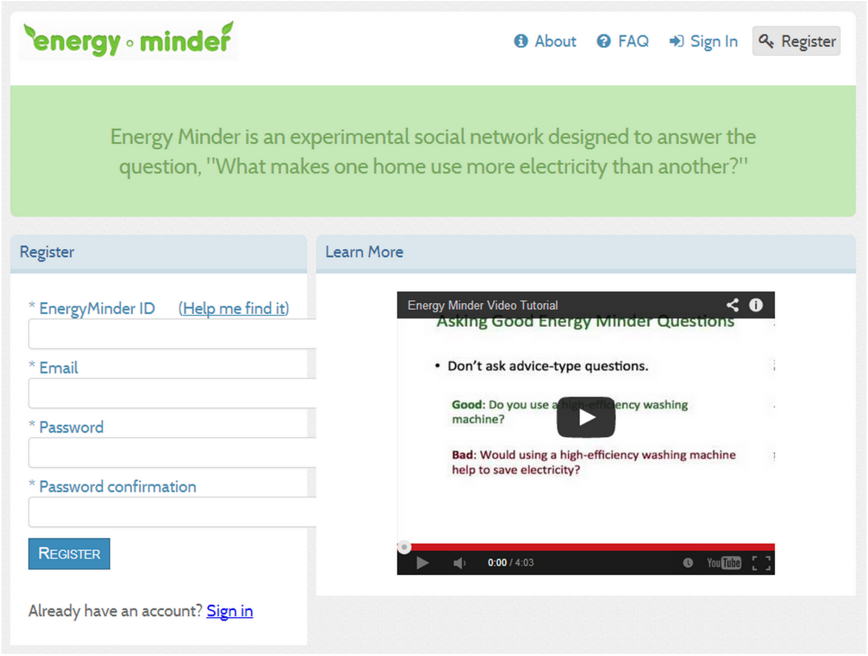

Figure D. Landing Page for the EnergyMinder site.

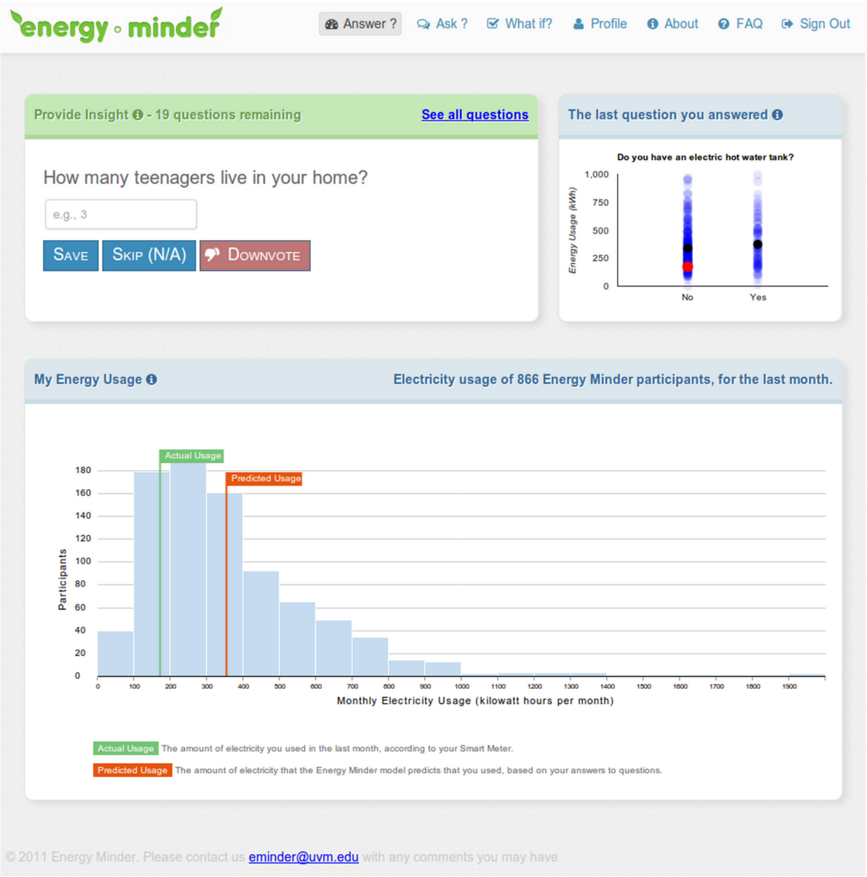

Figure E. Primary Survey Page for the EnergyMinder site.

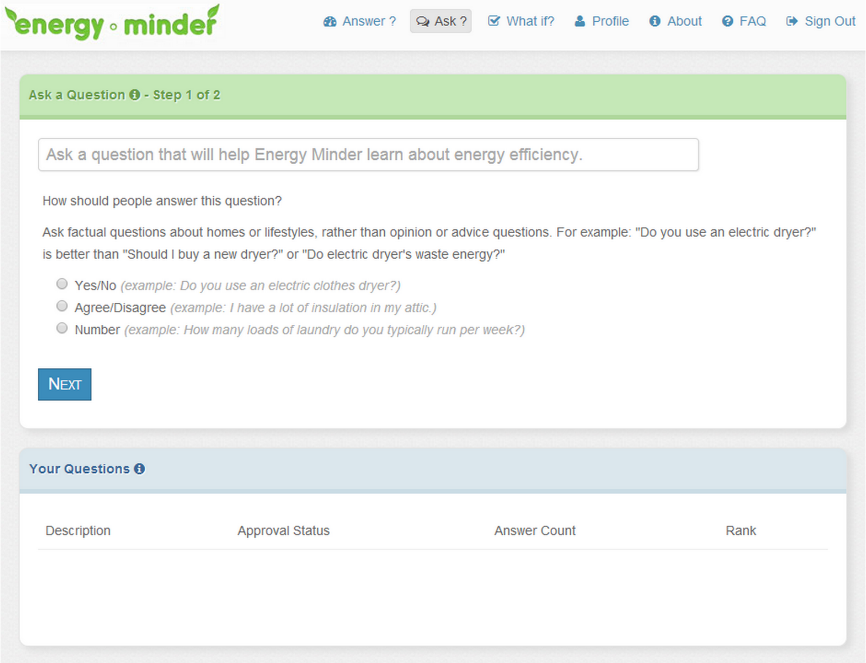

Figure F. Question Submittal Page for the EnergyMinder site.

Personal Savings

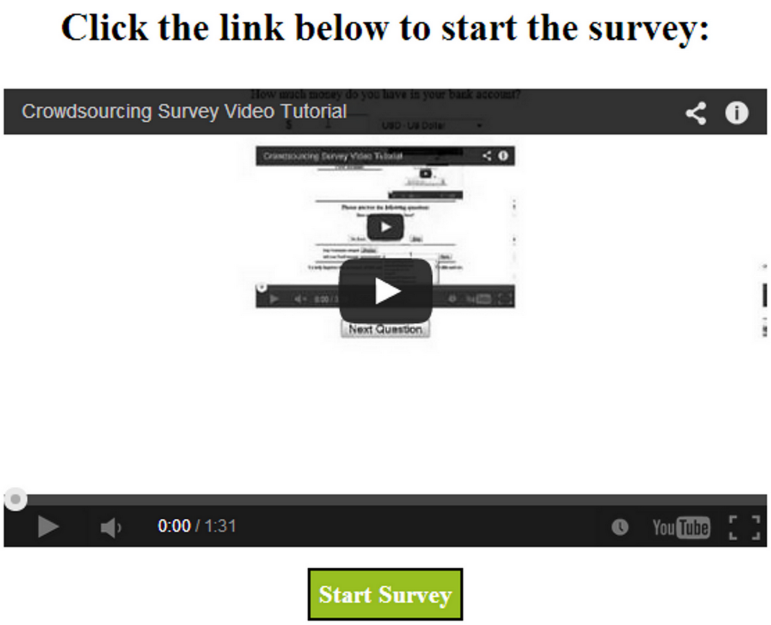

Figure G. Landing Page for the Personal Savings site.

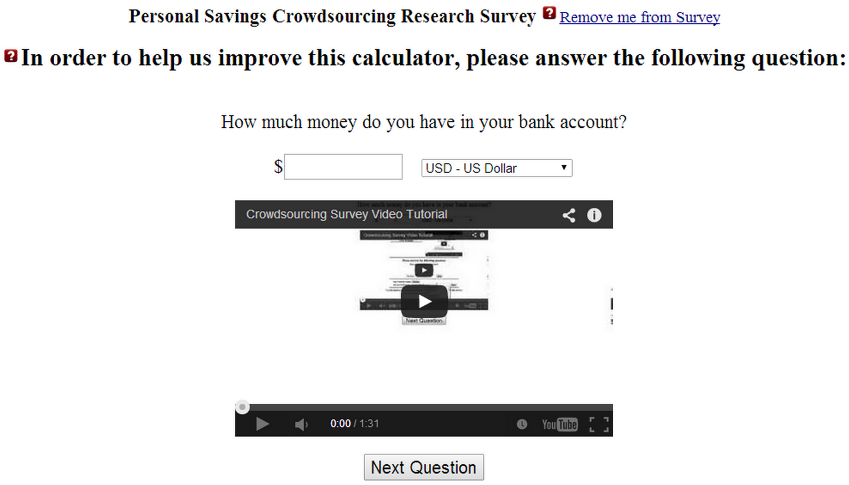

Figure H. Second Landing Page for the Personal Savings site.

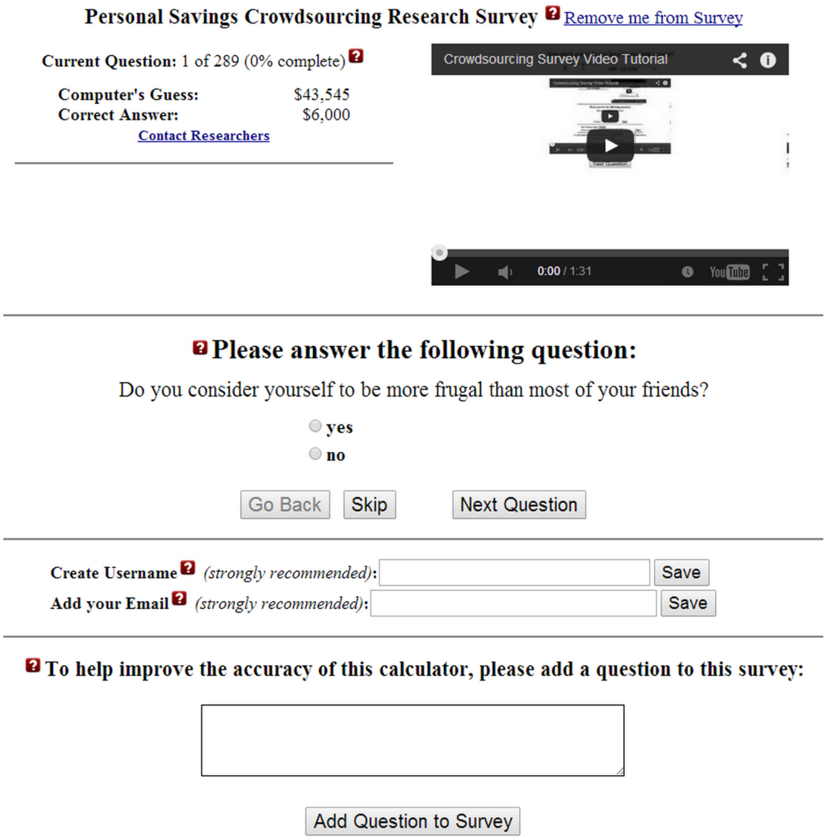

Figure I. Primary Survey Page for the Personal Savings site.
